# PACKMAN – A portable instrument to investigate space weather

**DOI:** 10.1016/j.ohx.2020.e00169

**Published:** 2020-12-26

**Authors:** Thasshwin Mathanlal, Abhilash Vakkada Ramachandran, Maria-Paz Zorzano, Javier Martin-Torres

**Affiliations:** a Group of Atmospheric Science, Department of Computer Science, Electrical and Space Engineering, Luleå, University of Technology, Luleå 97 187, Sweden; b Centro de Astrobiología (CSIC-INTA), Torrejon de Ardoz, 28850 Madrid, Spain; c Instituto Andaluz de Ciencias de la Tierra (CSIC-UGR), 18100 Granada, Spain; d School of Geosciences, University of Aberdeen, Meston Building, King's College, Aberdeen AB24 3UE, UK

**Keywords:** Space weather, Radiation, COTS, Open-source, Magnetic anomaly, Earth observation

## Abstract

PACKMAN (PArticle Counter k-index Magnetic ANomaly) is an autonomous, light and robust space weather instrument for operation within the subsurface, surface and atmosphere (as payload in stratospheric balloons) of the Earth. It has been designed using Commercial Off-The-Shelf (COTS) components to reduce the cost of each unit and to allow to have multiple units monitoring simultaneously at different sites and also incorporate an open-access citizen science approach. The hardware-core of each PACKMAN units, weights around 600 g and consumes about 500 mA of current at 12 V. PACKMAN has been deployed at multiple latitudes and altitudes ranging from stratospheric heights (corroborating its TRL8 maturity) to subsurface depths of around 1 km. The data from PACKMAN have been compared with the state-of-the-art ground-based observatories, and satellites and scientific observations have been documented. A 3-D network of PACKMAN units operating continuously around the globe, from the subsurface to the stratosphere, would help to improve the understanding of the space weather phenomena, and its implications on the climate and infrastructures. PACKMAN is also an excellent tool for education and outreach. This article outlines the building instructions of two types of PACKMAN units: PACKMAN-S for ground-based measurements and PACKMAN-B for stratospheric measurements aboard high-altitude balloons.

## 1 Specifications table

**Table:** 

Hardware name	PACKMAN [PArticle Counter k-index Magnetic Anomaly]
Subject area	• Environmental, Planetary and Agricultural Sciences
Hardware type	• Measuring physical properties and in-lab sensors • Field measurements and sensors • Electrical engineering and computer science
Open Source License	GNU General Public License (GPL) 3.0
Cost of Hardware	835 GBP
Source File Repository	https://data.mendeley.com/datasets/g86wh6k6dh/1

## 2 Hardware in context

Space weather, the weather phenomena invisible to the naked eye caused by solar wind, solar flares and cosmic ray particles coupled with the variability in the meteorological processes in the lower atmosphere [1] has a crucial impact on our planet. The Earth’s atmosphere is constantly bombarded by high energetic particles emanating from the Sun and outer space. Cosmic rays from distant galaxies also make it to the Earth’s atmosphere and are termed as Galactic Cosmic Rays (GCR). The dynamic interaction of these high energy particles with the Earth’s magnetic field is responsible for the space weather that prevails in the near-Earth space and the upper part of the Earth's atmosphere. One of the visible effects of space weather is observed as the colourful northern lights, Aurora Borealis and southern lights, Aurora Australis observed in the Northern and Southern latitudes respectively. Space weather has been attributed to impact various atmospheric processes, by altering the atmospheric chemistry and temperature [2], [3], [4], global electric circuit [5] and cloud formation [6], [7], and dynamics from the thermosphere down to the surface [8]. Space weather also impacts our modern infrastructure like power grids, satellites, high-altitude flights and space exploration [9]. Space weather can have a major impact on the economies [10], [11], [12], [13], [14], [15], [16] discusses space weather as an emerging natural hazard. Studies on forecasting space weather events to mitigate some disruptions to satellite operations and electric grids are elucidated in [17]. To date, there is a missing gap of information regarding the amount, energy, time variability, and type of space radiation (particles trapped in the Earth’s magnetic field; particles traveling into space during solar flares; and galactic cosmic rays, which are high-energy protons and heavy ions from outside our solar system) that reaches the lower layers of the atmosphere, as well as on its geographic and altitude distribution. Obtaining such information over a broader spatial and temporal resolution would enrich our understanding of the implications of space weather on infrastructures and climate. The purpose of this article is to describe in detail a low-cost scientific instrument for autonomous, and robust, field operation, which in this case can monitor radiation and environmental parameters at the subsurface, surface and atmosphere up to the stratosphere of Earth. To generate a long-time, multiple-site, open-access record of space radiation on Earth we have designed PACKMAN (PArticle Counter k-index Magnetic ANomaly): an open-source, autonomous instrument, with Commercial Off-The-Shelf (COTS) components. PACKMAN is a robust, light-weight instrument with sensors to monitor space radiation, magnetic disturbances and environmental parameters. PACKMAN collectively measures all the three types of space radiation with a Geiger counter providing a basic insight on the radiation environment at different altitudes and latitudes. PACKMAN has operated at different latitudes: 1) Space campus LTU, Kiruna, Sweden (67.84 °N, 20.41 °E, 390 m); 2) LTU Main campus, Luleå, Sweden (65.62 °N, 22.14 °E, 15 m); 3) the University of Edinburgh, United Kingdom (55.94 °N, 3.19 °W, 98 m); 4) Boulby Mine, United Kingdom (54.56 °N, 0.82 °W, 93 m and −1.1 km), 5) the University of Akureyri, Iceland (65.68 °N,18.12 °W, 23 m); and two PACKMAN units have been flown in balloons to the stratosphere: 6) Cordoba airport, Spain (37.84 °N, 4.84 °W, 90 m to 27 km); and 7) Esrange Space Center, Sweden (67.88 °N, 21.12 °E, 328 m to 27 km). An overview of PACKMAN and some scientific observations from the instrument are presented in [18].

## 3 Instrument description

The concept of utilization of COTS components and open-source software for the development of PACKMAN has been an important design consideration. This is critical to ensure the development of a low-cost, scalable network of PACKMAN’s. The design is easily reproducible with the components available in the local market. The low-cost approach ensures the development of PACKMAN with a minimum investment of resources, such that PACKMAN is globally affordable. PACKMAN is an instrument of its kind and competes for existing terrestrial instruments such as the uRAD environmental network [19]. uRAD is an Internet of Things (IoT) device chain that provides visual data representation of the radiation at nodes installed around the globe. uRAD monitoring network lacks magnetic field observation, and the data is not available in open format. PACKMAN combines a broader sensor suite under one roof with sensors to monitor space radiation, magnetic anomaly and environmental parameters such as temperature, humidity and pressure. Moreover, the data from PACKMAN would be available in an open format for public, universities and researchers around the world to access. PACKMAN can also be deployed aboard stratospheric balloons to study the interaction of energetic particles precipitating in the atmosphere at higher altitudes.

**Fig. 1:**
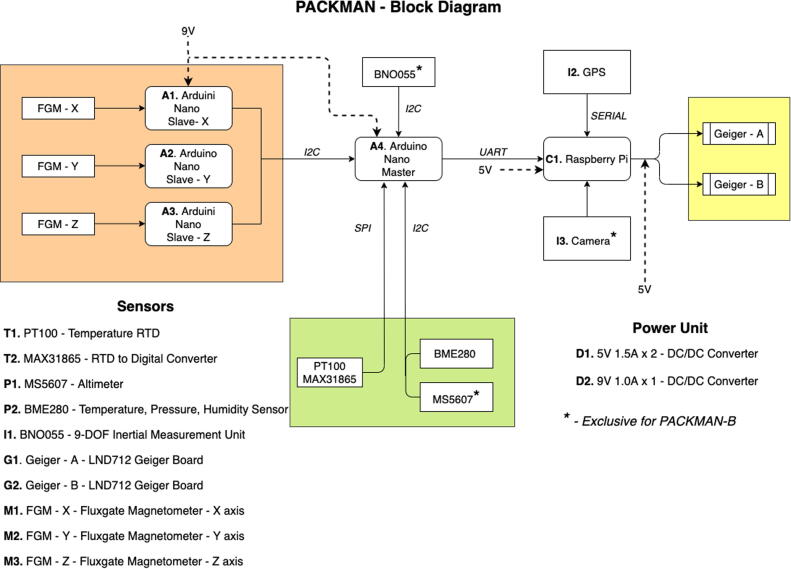
PACKMAN operational Block Diagram.

PACKMAN is available in two configurations – PACKMAN-S and PACKMAN-B. The former is the surface version which operates on a standard 12 V DC 2.5 A power supply and requires an active LAN (Local Area Network) internet connection to upload data to a cloud service. PACKMAN-B is deployed as a payload aboard stratospheric and high-altitude balloons and is powered by 10 AA-size FR6 batteries. It constitutes a piggyback payload and operates independently of other payloads on balloons. The two configurations are identical in construction with minor changes in configuration and software. Fig. 1 shows the block diagram elucidating the operation of PACKMAN.

As shown in the figure above, both the versions of PACKMAN have identical block design, except for the extra sensors such as the MS5607 altimeter, BN0555 Inertial Measurement Unit (IMU) sensor, Global Positioning System (GPS) sensor and camera. The sensors chosen for the PACKMAN have a suitable range of measurements needed for the scientific analysis, along with good accuracy and precision. They can be easily interfaced with the Arduino platform using either Inter-Integrated Circuit (I2C) or Serial Peripheral Interface (SPI) protocol and have excellent library support. Table 1 below shows the sensors used in PACKMAN along with their basic specifications such as range, accuracy along with the manufacturer and breakout board designer details. The sensors exclusive to PACKMAN-B such as the GPS and IMU does not provide direct scientific information and hence can be substituted with the best alternative.

**Table 1:** PACKMAN basic sensor specifications.

Sensor	ID	Physical variable	Range	Accuracy	Manufacturer	Breakout board design
LND712	G1 G2	Particle count (alpha/beta/gamma)	0–10 mSv/hr	0.01 uSv/hr	LND, INC	Sparkfun
FG-3+	M1 M2 M3	Magnetic Field	± 50,000 nT	±15 nT	FG Sensors	NA
BME280		Temperature	−40 to 85 °C	±1.25 °C	Bosch	Adafruit
	P2	Humidity	0 to 100% RH	± 3%		
		Pressure	300 to 1100 mbar	±1 mbar		
MS5607	P1	Pressure	10–1200 mbar	0.054 mbar	TE Connectivity	
PT100/MAX31865	T1 T2	Temperature	−200 °C to 600 °C	0.5 °C	Maxim Integrated	Adafruit

Considering long-term commissioning of PACKMAN, COTS sensors can retire from the market and would need to be substituted with sensors of equivalent configuration. Other sensors with similar specifications can be replaced for in PACKMAN. The specifications of the sensors listed meet the exact needs of the instrument. In the event of such replacement, the users have to update the Arduino codes based on the sensor used and the data format, units and data frequency have to be maintained as that of the original code to preserve uniformity and conformance. The raw Geiger Counts Per Minute (CPM) are extracted from the sensor, and their conversion to equivalent radiation dosage (uSv/h) would depend upon the type of Geiger tube used. LND712 can measure alpha, beta and gamma radiation collectively with the capability to detect alpha particles above 3 MeV in energy, beta radiation above 50 KeV and X-Ray and Gamma radiation above 7 KeV. The full range of measurement counts of the LND712 Geiger tube is 1 Count Per Minute (CPM) to 10,000 Counts Per Second (CPS). The conversion factor of LND712 equates to 108 CPM per uSv/h. Information on commercial Geiger tubes available in the market along with their specifications and conversion factor has been detailed in [20].

The Raspberry Pi used on the PACKMAN-B version is the model A+, while the PACKMAN-S version uses the Raspberry Pi 3B + Model. The PACKMAN-B does not need real-time data access, and hence it is fitted with a low power consuming model A + which lacks network connectivity. PACKMAN-S needs to have real-time data access and therefore requires a Raspberry Pi with RJ45 LAN connectivity. PACKMAN-B can be ideally used as PACKMAN-S by disabling camera at the cost of no real-time data dissemination. In contrast, the latter cannot be used as a Balloon payload as it lacks flight sensors such as altimeter and IMU. PACKMAN-B uses GPS to create a Network Time Protocol (NTP) server using the National Marine Electronics Association (NMEA) data which provides timestamp to the sensor measurements with a few hundred-millisecond accuracies. The Pulse Per Second (PPS) enabled GPS unit enables precise measurement of the start of the second and enables accuracy to few nanoseconds. In the case of PACKAMAN-S, the default NTP configuration of Raspbian operating system is used for timekeeping as there is network access available through LAN connection.

PACKMAN units operate with Raspberry Pi as the central processing computer. The Raspberry Pi provides excellent flexibility to interface sensors and Arduino microcontrollers. The cost of the Raspberry Pi computers is comparatively lower to the competitive System on Chip (SoC) computers available in the commercial market. The increased technical support available online and global availability is one of the driving factors to use Raspberry Pi in the PACKMAN units. Arduino is used as the preferred microcontroller, owing to similar reasons of the Raspberry Pi such as low-cost, flexibility, global availability, more extensive technical support online. The PACKMAN uses Raspbian operating system on the Raspberry Pi burnt on an industrial-grade microSD card. These industrial-grade microSD cards are built to withstand advanced wear levels and have data integrity protections features and are robustly durable to withstand harsh −40 °C to 85 °C temperature operable environments. The Raspbian OS is pre-configured with the python scripts, launch scripts and needed system configuration such that the OS can be used straight out of the box. However, the python scripts, shell scripts and configuration files are also attached with the article in case users would like to upgrade any component of PACKMAN. PACKMAN-S uses the ownCloud service to upload the data to the cloud. Users can create their ownCloud workspace and upload the data from the PACKMAN to their workspace, which can later be shared across research groups around the world. The data will be available for the global research community, as long as the instrument team is properly recognized in the references and data sources.

**Fig. 2:**
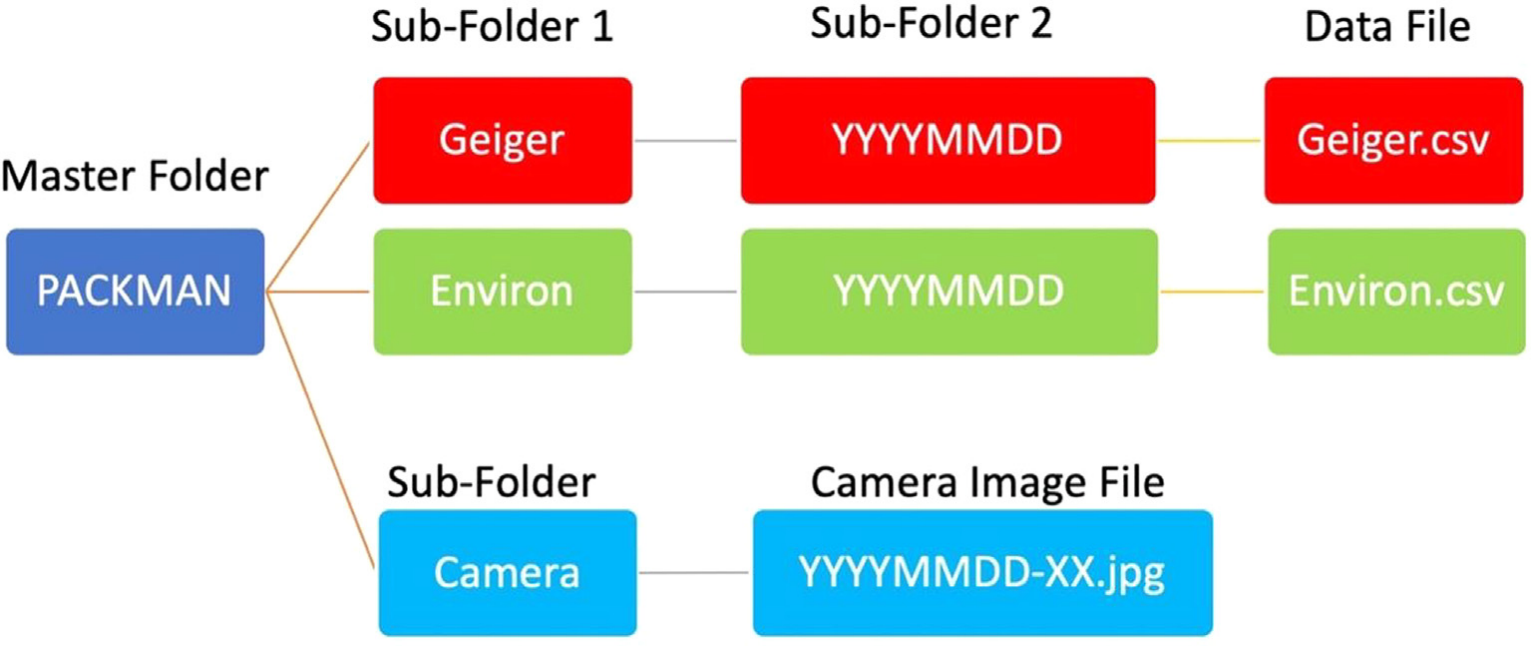
PACKMAN data folder architecture.

The data from the PACKMAN is generated in date-time based folder hierarchy. The data structure is explained in the following flowchart, Fig. 2. Having a uniform data format improves readability and simplifies processing of the data and enables comparison of data from different PACKMAN units operating around the globe. ISO 8601-1:2019 based date and time representation format has been used in PACKMAN.

**Fig. 3:**
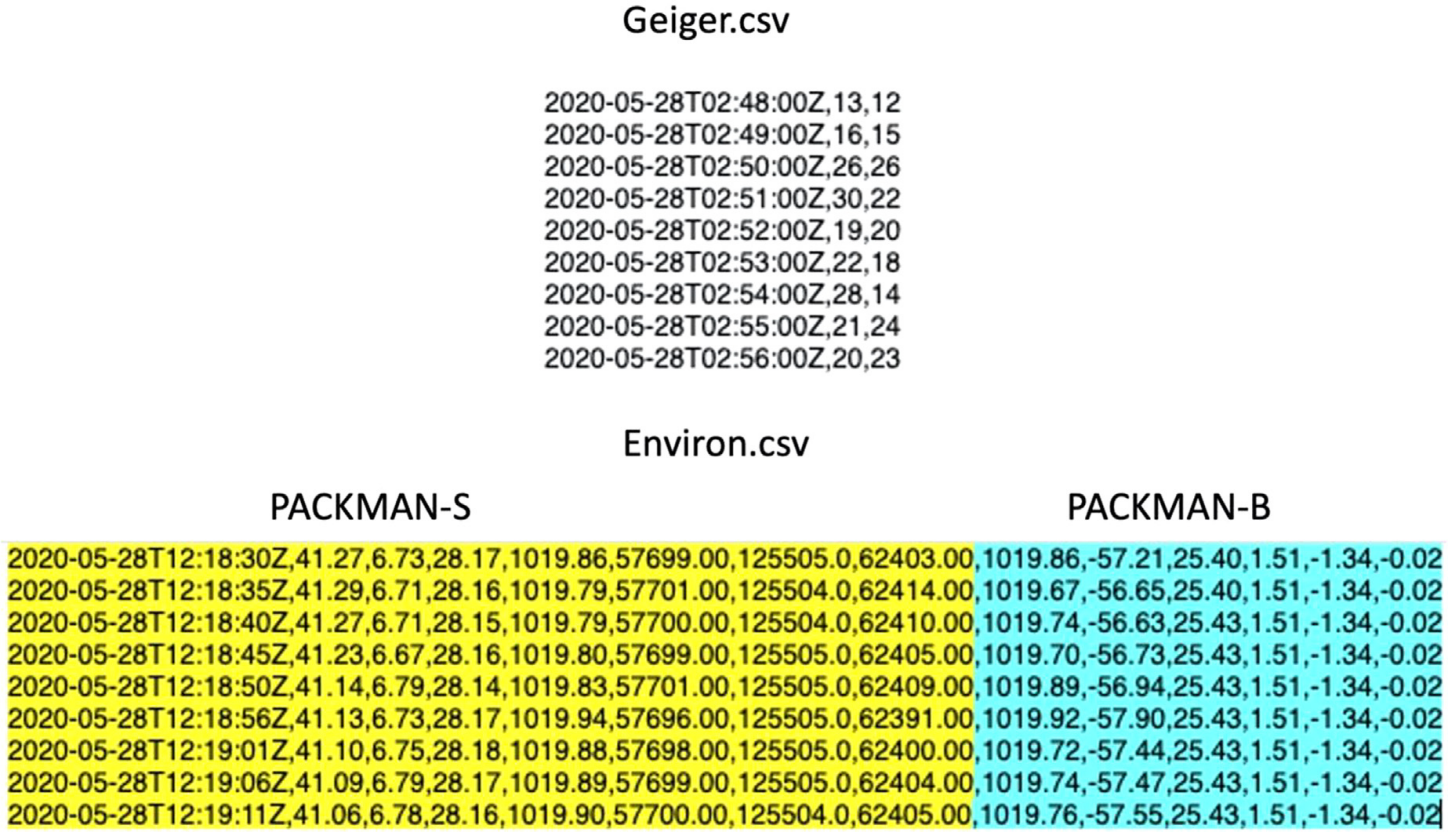
Sample data file – Geiger data (top) and Environmental data (bottom) generated by PACKMAN-B. The yellow highlighted part of the data shows the typical data output from PACKMAN-S. The blue highlighted portion of the information is exclusively made by PACKMAN-B in addition to the yellow part. The parameter headings of the corresponding data entries are shown in Table 2.

The Camera data folder exists only for PACKMAN-B version. The parameters measured along with their respective units of measurement are saved in the Environ.csv and Geiger.csv files. The order in which the variables are saved is tabulated as follows in Table 2. A quick look at the sample data file from PACKMAN-B is shown in Fig. 3. The parameters marked in blue are measured by PACKMAN-B exclusively. Each line in the file ends with a new line character.

**Table 2:** The data format of PACKMAN data files.

PACKMAN units have a potential for following applications

• Real-time dissemination of particle counts, magnetic field and environmental data.
• Monitor natural radiation sources
• Compare with Earth climate observations
• Education and Public awareness – Citizen Science approach

## 4 Design files

The source to the design files can be found in the Mendeley repository> https://data.mendeley.com/datasets/g86wh6k6dh/1 > Design Files.

**Table:** 

Design file name	File type	Open source license	Location of the file
PACKMAN_BASE	.STL	CC BY 4.0	Mendeley > Data > CAD_Files
PACKMAN_BOX	.STL	CC BY 4.0	Mendeley > Data > CAD_Files
PACKMAN_LID	.STL	CC BY 4.0	Mendeley > Data > CAD_Files
PACKMAN_DECK_TOP	.STL	CC BY 4.0	Mendeley > Data > CAD_Files
PACKMAN_DECK_BASE	.STL	CC BY 4.0	Mendeley > Data > CAD_Files
PACKMAN_GEIGER_SPACER	.STL	CC BY 4.0	Mendeley > Data > CAD_Files
PACKMAN_MAGNETOMETER_HOLDER	.STL	CC BY 4.0	Mendeley > Data > CAD_Files
PACKMAN_MAGNETOMETER_SLAVE	.INO	GNU General Public License (GPL) v3	Mendeley > Data > Arduino_Programs
PACKMAN_S_MASTER	.INO	GNU General Public License (GPL) v3	Mendeley > Data > Arduino_Programs
PACKMAN_B_MASTER	.INO	GNU General Public License (GPL) v3	Mendeley > Data > Arduino_Programs
PACKMAN_GEIGER_PYTHON	.PY	GNU General Public License (GPL) v3	Mendeley > Data > Python_Scripts
PACKMAN_ENV_PYTHON	.PY	GNU General Public License (GPL) v3	Mendeley > Data > Python_Scripts
PACKMAN_B_CAMERA_PYTHON	.PY	GNU General Public License (GPL) v3	Mendeley > Data > Python_Scripts
PACKMAN_B_FILE_TRANSFER	.PY	GNU General Public License (GPL) v3	Mendeley > Data > Python_Scripts
PACKMAN_S_CRONTAB_CONFIG	.TXT	GNU General Public License (GPL) v3	Mendeley > Data > Python_Scripts
PACKMAN_B_CRONTAB_CONFIG	.TXT	GNU General Public License (GPL) v3	Mendeley > Data > Python_Scripts
PACKMAN_B_MOUNT	.SH	GNU General Public License (GPL) v3	Mendeley > Data > Shell_Scripts
PACKMAN_S_CONFIG	.SH	GNU General Public License (GPL) v3	Mendeley > Data > Shell_Scripts
PACKMAN_B_OS	.IMG	GNU General Public License (GPL) v3	Mendeley > Data > Operating_System
PACKMAN_S_OS	.IMG	GNU General Public License (GPL) v3	Mendeley > Data > Operating_System

### 4.1 Hardware

PACKMAN_BASE, is the CAD file of the 3D printed PACKMAN BASE that mounts all the components.
PACKMAN_BOX, is the 3D printed CAD file of the PACKMAN enclosure.
PACKMAN_LID, is the 3D printed CAD file of the PACKMAN enclosure lid.
PACKMAN_DECK_TOP, is the 3D printed CAD file of PACKMAN, that holds the magnetometer Arduino slave PCB and Arduino Nano Master PCB.
PACKMAN_DECK_BASE, is the 3D printed CAD file of PACKMAN, that holds the Raspberry Pi, Power PCB and GPS unit.
PACKMAN GEIGER_SPACER, is the 3D printed CAD file of PACKMAN, that is used to mount the Geiger boards.
PACKMAN_MAGNETOMETER_HOLDER, is the 3D printed CAD file of the PACKMAN fixture used to mount the magnetometers on the orthogonal axis.

### 4.2 Software

PACKMAN_MAGNETOMETER_SLAVE, is the Arduino program for the three Arduino Nano slaves.
PACKMAN_MASTER, is the Arduino program for the Arduino Nano master.
PACKMAN_GEIGER_PYTHON, is the python code to capture data from the Geiger sensors.
PACKMAN_GEIGER_ENV_PYTHON, is the python code to capture data from the environmental sensors.
PACKMAN_CAMERA_PYTHON, is the python code to capture data from the camera.
PACKMAN_FILE_TRANSFER, is the python code that executes the data transfer to the USB drive.
PACKMAN_CRONTAB_CONFIG, is a text file containing the CRONTAB entries that initiate the python scripts on the Raspberry Pi.
PACKMAN_MOUNT, is the shell script that works along with PACKMAN_FILE_TRANSFER python script.
PACKMAN_B_OS, is the image file of the Raspbian OS pre-configured to operate PACKMAN-B.
PACKMAN_S_OS, is the image file of the Raspbian OS pre-configured to operate PACKMAN-S.

## 5 Bill of materials

The full bill of materials is shown in Table 3. The materials needed exclusively for PACKMAN-B are colour coded in blue and materials solely needed for PACKMAN-S are colour coded in green. Standard materials are colour coded in yellow. The sensors and critical components are labelled with ID number corresponding to the Block Diagram, shown in Fig. 1. The source links to the components can be found in the Bill of Materials (BOM) excel document uploaded to the Mendeley repository > Data > Bill of Materials. The prices mentioned are indicative and not considering the Minimum Order Quantity (MOQ).

**Table 3:** Bill of Materials of PACKMAN.

## 6 Build Instructions

This section is split into two parts – hardware building and software configuration, respectively. The former part details the 3D printed component assembly, PCB assembly and wiring. The latter elucidates the software configuration needed to operate PACKMAN.

### 6.1 Hardware building

The hardware building for PACKMAN-B and PACKMAN-S are identical except for few more added sensors, file transfer PCB and power connection variation in PACKMAN-B. PACKMAN-S operates using a wall-mounted power supply with a DC barrel jack and hence uses 2 mm barrel connector fixed to a male DB-9 connector. In case of PACKMAN-B, the unit operates on AA-size batteries connected in series through a battery holder. The battery holder clip is connected to the male DB-9 connector. Radio Frequency (RF) interference from wall mounted power supplies do not pose a threat to the sensor operations of PACKMAN owing to the presence of linear regulator built-in the Arduino boards which reduce the switching noise present in the output of wall mounted supplies.

### 6.2 Step-1: 3D printing

The 3D printed components used in PACKMAN are 3D printed in PolyLactic Acid (PLA) material with a sufficient infill density (greater than80%), for increased strength. Appropriate support settings are used depending upon the 3D printer model. The 3D printed components have no overhanging structures except for the tubular PACKMAN_MAGNETOMETER_HOLDER component and some windows in the PACKMAN_BOX component. Support is needed only for these two components and ensures the structures are removed carefully and edges smoothened with files and sandpaper. The CAD file of the 3D printed camera enclosure is not attached with the design files. Depending upon the Raspberry Pi camera used, suitable 3D printed enclosure can be used.

### 6.3 Step-2: PCB assembly

**Fig. 4:**
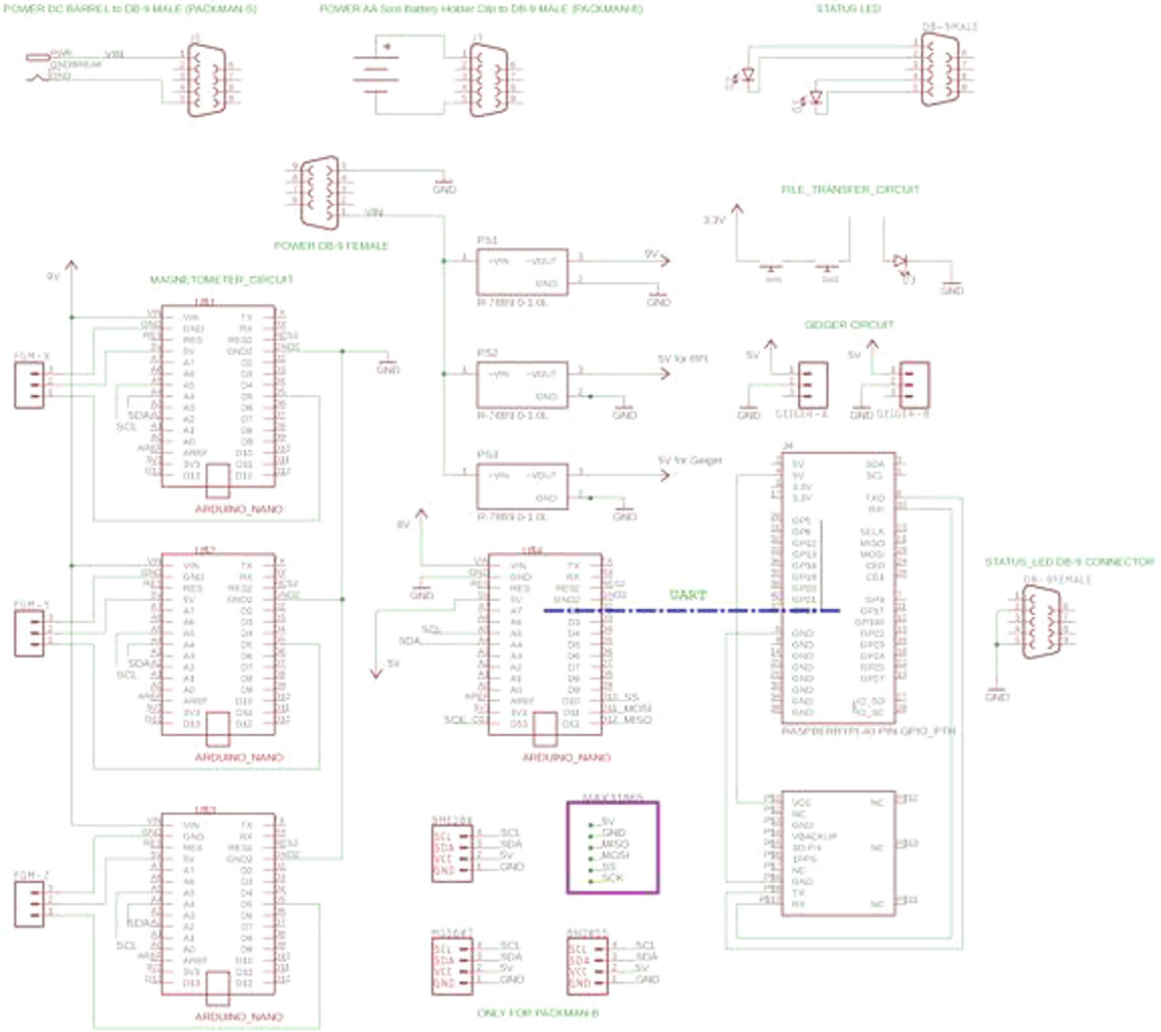
PACKMAN Circuit Connections.

PACKMAN has five custom PCB’s - Four mini 35x50mm size PCB from Sparkfun and two 81x51mm size PCB from Adafruit. An Adafruit T-Cobbler Plus 2x20 PCB is used to connect the GPS, status LED and Geiger counter PCB’s. A 35x50mm size PCB is connected to the Raspberry Pi housing two buttons and an LED, which are used to initiate the data transfer from the PACKMAN-B to the USB drive. The full circuitry connection of PACKMAN is shown below in Fig. 4.

### 6.4 Step-3: wiring

Multistrand copper wires of gauge 24 AWG are used in wiring the individual circuits. Use of appropriate colour coding can help to trace the wiring between circuits. (e.g., red for positive line, black for ground line, green for I2C clock line (SCL) and blue for I2C data line (SDA). Such methodical colour coding can help to trace conductivity between components during troubleshooting of bad soldering joints. Tinting the multi-strand copper wires before soldering to the PCB prevents bad soldering joints. Wires between PCB’s and magnetometers are provided sufficient length, to facilitate easy assembly. The power DB-9 and LED status DB-9 pins are not soldered at this time as the wires have to go out through the 3D printed PACKMAN_BOX. DB-9 pins are used as they offer fool-proof (poke-yoke) capability, and wrong polarity connections cannot be made.

### 6.5 Step-4: assembly

Once the individual components are soldered and wired onto the respective PCB’s, the Raspberry Pi, Arduino, GPS and soldered, PCBs can be assembled onto the 3D printed Deck 1 and Deck 2. The assembly is done in the bottom to top approach with Deck 1 containing the PCB of the magnetometer Arduino slaves and the Master Arduino PCB. The Deck 2 Is fitted with Power PCB, Raspberry Pi, and GPS. Deck 2 would also house the IMU sensor and pressure sensor in case of PACKMAN-B. The orientation of the IMU sensor is crucial for calibrating the PACKMAN-B unit. Ensure the IMU sensor is soldered to the PCB with the axis orientation aligned with the fluxgate magnetometer XYZ orientation which is mentioned in the following section. Appropriate spacers are used to mount the PCB’s, Raspberry Pi and GPS on to the 3D printed decks. Four M4x50 bolts and nuts are used to secure the decks one above the other to create the deck assembly.

The Geiger counter PCB is then mounted onto the PACKMAN_BASE 3D printed part towards the edge. Four M4 holes are drilled on the base, and M4x60 bolts with PACKMAN_GEIGER_SPACER is used to stack the Geiger counters one above the other. The Deck assembly is then fixed next to the Geiger assembly by drilling the base from the bottom and attaching with four M4x20 bolts and nuts.

The PACKMAN_MAGNETOMETER_HOLDER is glued using Universal instant glue to the extreme corner of the PACKMAN base on the farthest extent from the Geiger counter. The three fluxgate magnetometers are then inserted into the 3D printed PACKMAN_MAGNETOMETER_HOLDER in proper axis configuration. The vertical stem constitutes the Z-Magnetometer, and the Y-Magnetometer is fixed along the longer side of the PACKMAN_BASE.

Cable ties are used to route the wires to avoid dangling of the wires properly. Connect the USB cable between the Raspberry Pi and the Arduino Master Nano present in the lower deck. Attach the Camera ribbon cable to the Raspberry Pi camera socket. The PT100 probe wire is fixed to the sensor breakout board. The Raspberry Pi micro USB cable can be plugged into the Raspberry Pi power socket.

**Fig. 5:**
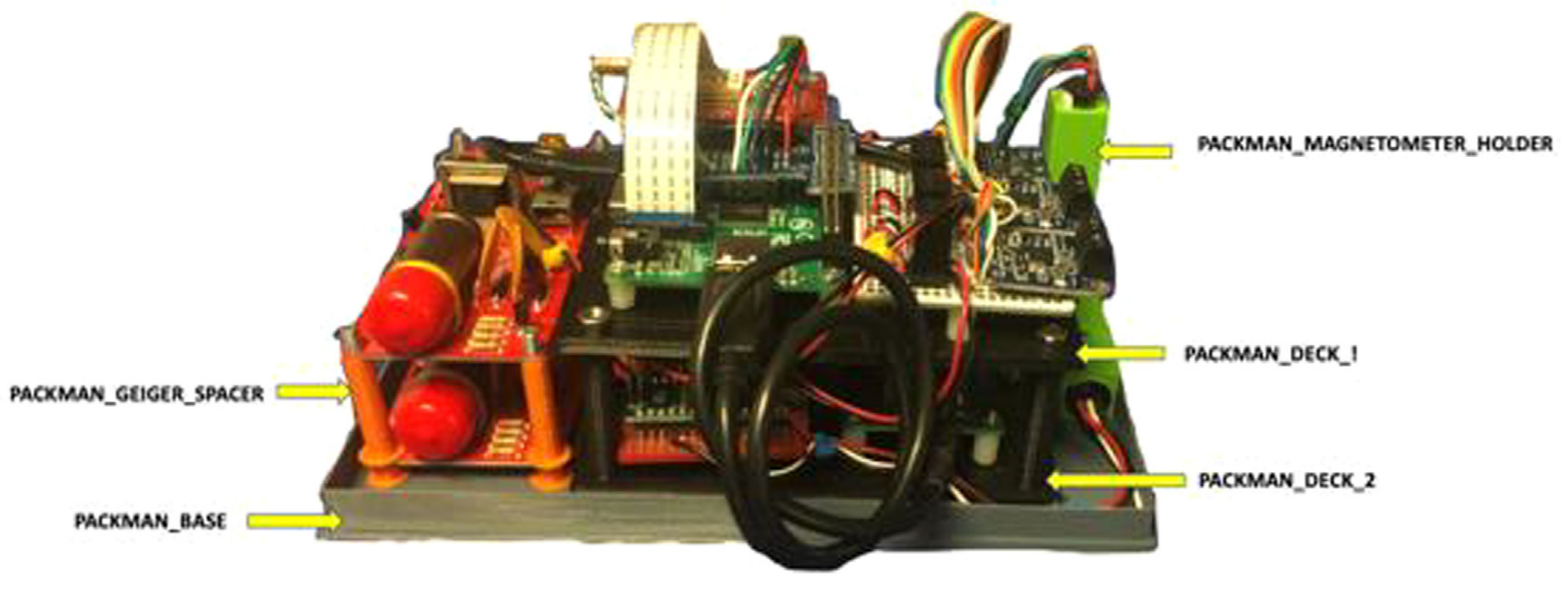
PACKMAN unit assembled and 3D printed components marked.

The overall assembly of the PACKMAN unit would resemble Fig. 5 below without the PACKMAN_BOX enclosure.

**Fig. 6:**
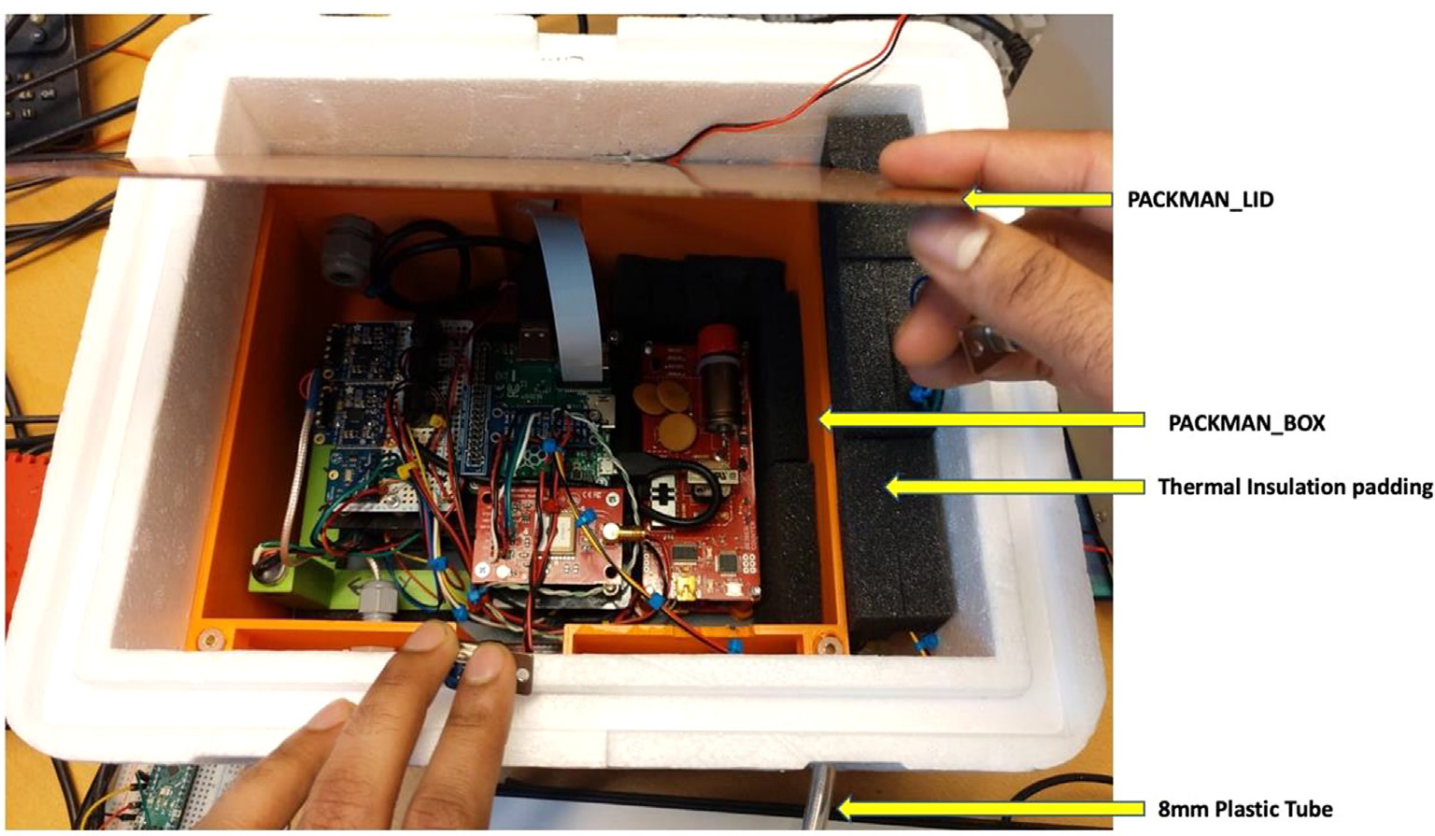
PACKMAN unit enclosed in the Styrofoam box.

The entire PACKMAN_BASE is then placed into the PACKMAN_BOX 3D printed enclosure, which in turn is placed into a Styrofoam box. A Styrofoam box of 250 × 200 × 150 mm inner dimension has been used to house PACKMAN units. The Camera ribbon cable, status LED wires, power wires and PT100 are taken out through the respective windows, and the DB-9 connectors of the status LED and power are soldered. The PT100 probe wire, status LED and power wires are fed through M3 cable clamps attached to the 3D printed enclosure. A M5 cable clamp is used to fix an 8 mm tube which is used to equalize the pressure inside and outside the Styrofoam box of PACKMAN-B during flight. In the case of PACKMAN-S, this tube is used to provide airflow into the PACKMAN unit. The PACKMAN BOX is then placed into the Styrofoam box, as shown in the figure, Fig. 6 below. Padding is provided with pieces of insulation foam to prevent the PACKMAN_BOX from moving within the Styrofoam. The insulation sheet also offers excellent thermal isolation for the temperature sensitive fluxgate magnetometers. The external PCB in PACKMAN-B is kept under these insulation pieces before flight.

Holes are made in the Styrofoam box to route the PT100 probe, status LED and camera out of the Styrofoam box in PACKMAN-B. In case of PACKMAN-S, holes are made on the Styrofoam box to route PT100 probe, status LED and RJ45 LAN cable. It is up to the user’s discretion to decide the layout of the hole positions on the Styrofoam box to route the cables.

### 6.6 Software configuration

The system configuration of PACKMAN-B and PACKMAN-S are explained in the following section. The operating systems for both the versions of PACKMAN are pre-configured and are attached in the Mendeley repository. PACKMAN-B OS can be used as such with no configuration settings needed. In the case of PACKMAN-S, a configuration utility is designed to facilitate the quick and easy setup of PACKMAN-S.

Setting up PACKMAN-B

1. Download the PACKMAN_B_OS from the Mendeley repository using the link mentioned in the Design file section.
2. Use Win32DiskImager software to write the downloaded OS to the memory card from the Windows operating system. In case of Linux or Mac operating system, the following commands are executed in terminal to write the OS to the SD card.
  **fdisk -l**

The command would list the mounted drives and note down the sdX for the memory card connected.

**dd bs = 4 M if = PACKMAN_B_OS.img of=/dev/sdX**

The command writes the contents of the img file to the SD card.

3. After the memory card is written with the OS, eject the memory card and insert into the Raspberry Pi SD card slot.

### 6.7 Setting up PACKMAN-S

1. Download the PACKMAN_SURFACE_OS from the Mendeley repository using the link mentioned in the Design file section.
2. Use Win32DiskImager software to write the downloaded OS to the memory card from Windows operating system. In case of Linux or mac operating system, the following commands are executed in terminal to write the OS to the SD card.
  **fdisk -l**

The command would list the mounted drives and note down the sdX for the memory card connected.

**dd bs = 4 M if = PACKMAN_B_OS.img of=/dev/sdX**

The command writes the contents of the img file to the SD card.

**Fig. 7:**
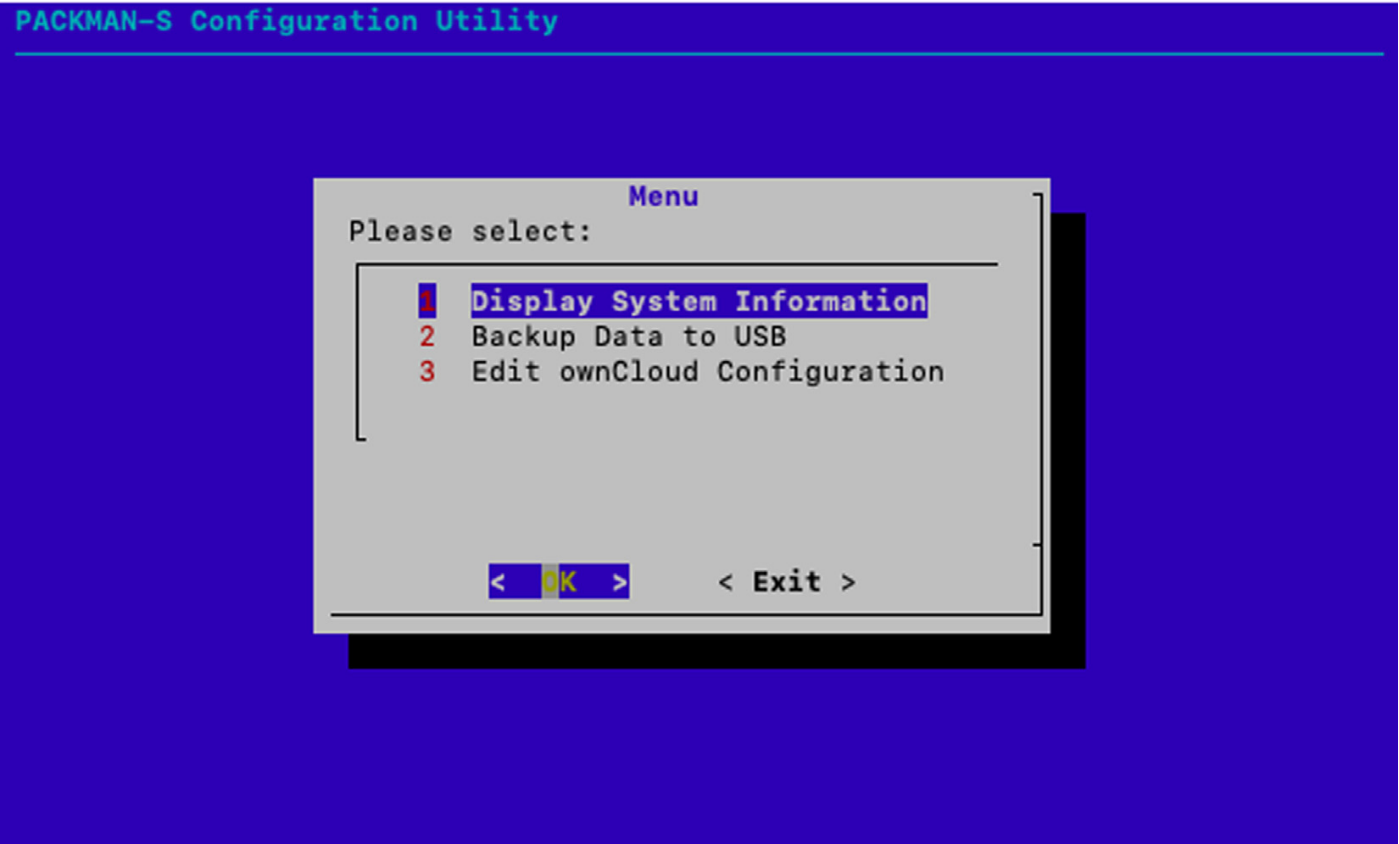
PACKMAN-S configuration utility home page.

1. After the memory card is written with the OS, eject the memory card and insert into the Raspberry Pi SD card slot.
2. Connect the LAN cable of the Raspberry Pi to the ethernet port of a desktop computer or laptop.
3. Power the PACKMAN unit with a 12 V DC supply connected to the Power DB-9 pin and wait for a couple of minutes.
4. Use a ssh terminal app such as PuTTY on a windows-based machine or the inbuilt terminal in Linux and mac computer.
5. Type ssh packman@packman-s.local to login to the PACKMAN-S terminal. Login with the following credentials:
  **Login:** packman
  **Password:** packman123
6. After the system is logged in, type **sudo packman-config**. This opens the PACKMAN-S configuration utility. The home screen of the configuration utility is shown as follows in Fig. 7. The configuration utility can be used to view some critical system information such as the Raspbian version, Linux kernel version and available memory space, as shown in Fig. 8. The utility can also be used to perform a data backup to a USB drive.

**Fig. 8:**
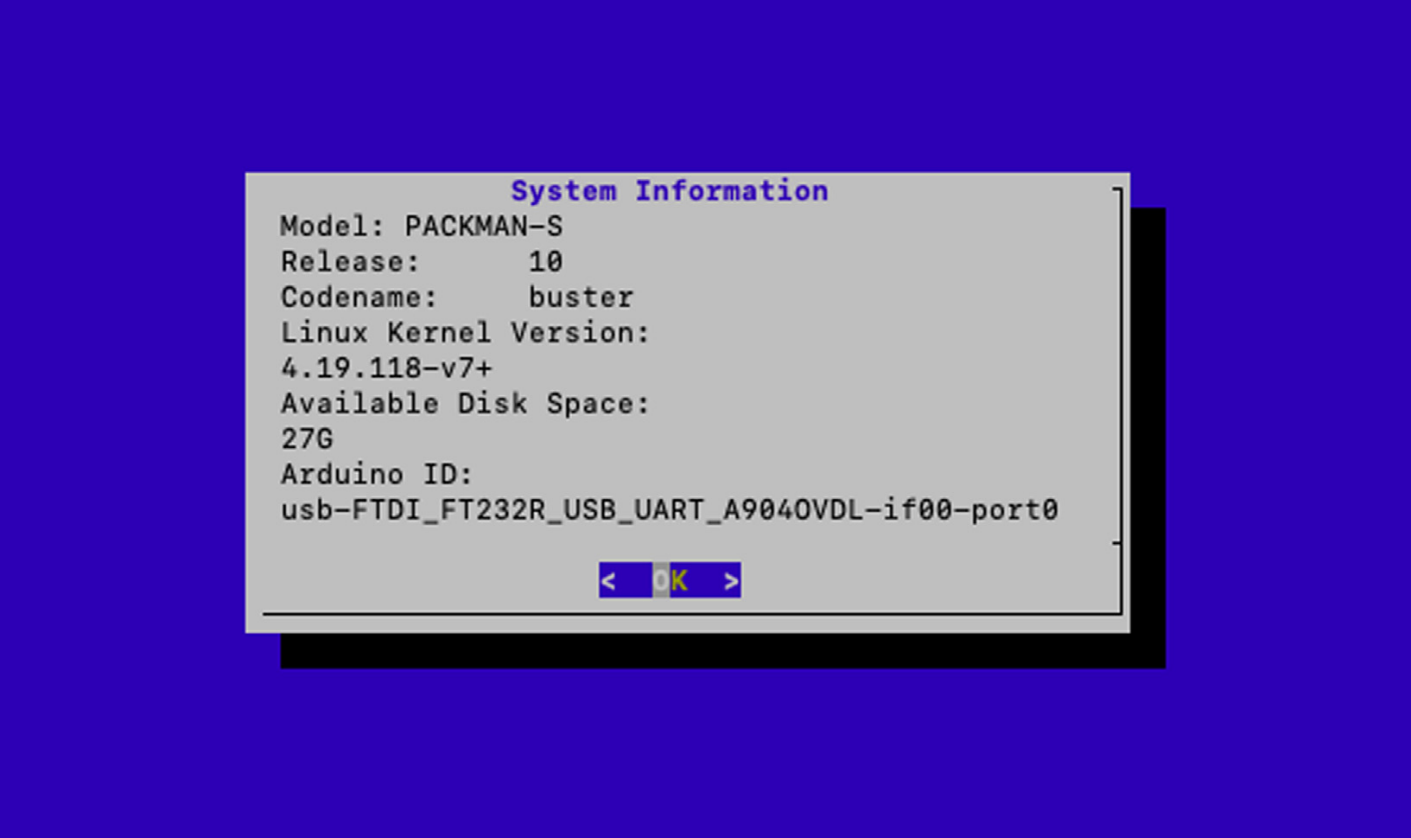
PACKMAN-S system information screen.
7. Edit the ownCloud configuration using the third option in the home screen. The options available in this window are shown in Fig. 9 as follows:

**Fig. 9:**
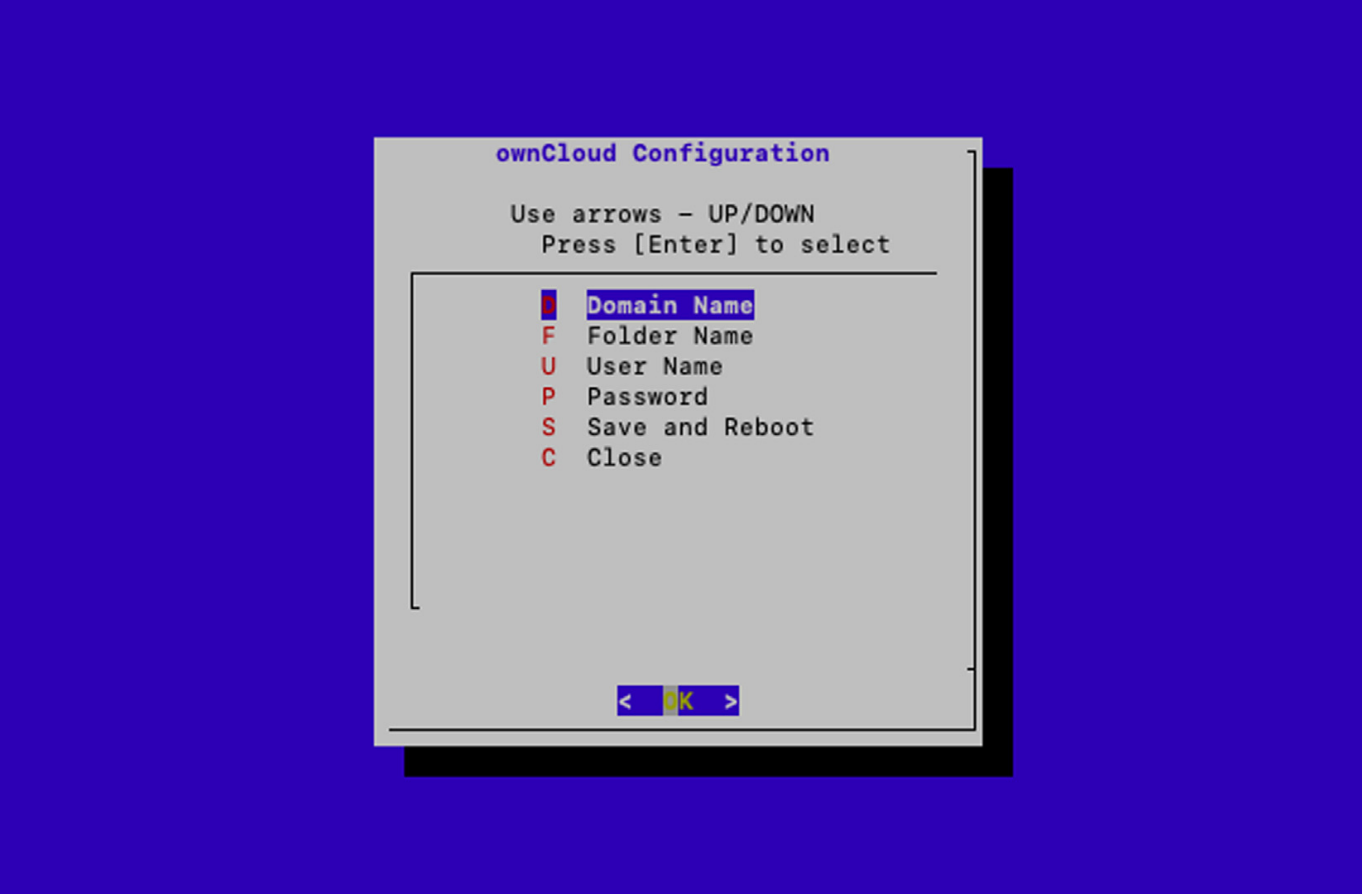
PACKMAN-S ownCloud configuration page.

Fill the configuration details one after the other and finally press Save and Reboot. The shortcut alphabets can also be used to enter the respective configuration details.

8. Once the system is rebooted, the PACKMAN-S is ready for operation.

## 7 Operation Instructions

### 7.1 PACKMAN-S operation

1. Connect the LAN cable and a 12 V DC 2.5 A adaptor to the PACKMAN-S unit.
2. Once, the status LED blinks green, and the PACKMAN-S has begun operation.
3. To view the data, login into the ownCloud workspace from any computer. The data is uploaded in every one-hour interval.

### 7.2 PACKMAN-B operation

PACKMAN-B operates on a DC power supply from 12 V to 18 V. AA-size batteries in series are used to power the PACKMAN-B. 10 AA-size FR6 batteries can provide an operational time of around 8 h for PACKMAN operation. Lithium-based AA size batteries are preferred as they are about 30% lighter than the regular alkaline batteries and have a better performance at sub-zero temperatures. PACKMAN-B operation is very straightforward. A 32 GB USB drive is all that is needed to recover the data from PACKMAN-B after the flight.

### 7.3 Before Launch

Just before the launch, the AA batteries are fixed in the battery holder, and the connector clip is secured to the battery holder. PACKMAN-B takes 5 min to begin operation. Ensure that PACKMAN-B is kept in an open environment (outside the building) during these 5 min. The 5-minute waiting time is given for the GPS to get a fix and also to calibrate the BNO055IMU sensor. The calibration sequence is performed as follows, as shown in Table 4. Ensure that the PACKMAN-B Styrofoam lid is held securely when performing the calibration procedure, to avoid the contents from dropping down.

**Table 4:** PACKMAN-B calibration instructions.

**Magnetometer Calibration** Move the PACKMAN-B, as shown in the pattern in one full cycle. The long side of the Styrofoam box has to face the user irrespective of the direction.	
**Accelerometer Calibration** Place the PACKMAN-B in these six stable positions for 2 s each. The orientation is such that in the first position, the top of the Styrofoam box faces to sky marking the z-direction (blue) and the long side is oriented along the Y-axis (green) and x-axis (red) is perpendicular to the orientation of the long side.	
**Gyroscope Calibration** Place the PACKMAN-B on a stable, flat position for about 5 s.	

PACKMAN-B will turn on after 5 min, and it can be verified by the LED blinking either red or green. The calibration status of PACKMAN can be obtained in the data file. The BNO055 sensor used returns a value between 0 and 3 (0 representing uncalibrated and 3 with best calibration). Proper calibration procedure followed should return a value greater than 0.

### 7.4 Data retrieval

With the payload recovered after the flight, the data can be transferred from the microSD card of PACKMAN-B to the USB drive by using the PCB with two pushbuttons. Table 5 illustrates the procedure to be adopted to initiate the data transfer to the USB drive.

**Table 5:** PACKMAN-B Instructions to retrieve data from the microSD card.

1. Remove the Styrofoam lid from PACKMAN-B and disconnect the battery connector from the battery holder.	
2. Disconnect the status LED DB-9 pin and lift the PACKMAN_LID to reveal the inner PACKMAN_BOX 3D printed enclosure.	
3. Remove the black insulation material to reveal the external PCB with the two buttons and a LED.	
4. Disconnect the USB wire connected to the Raspberry Pi. Ensure that the camera ribbon wire is not damaged during this step. Connect a 32 GB USB drive to the USB port of the Raspberry Pi. The space is very confined and hence use a USB drive that is not very long. A small USB drive, like the one mentioned in the Bill of materials, is preferred.	
5. Connect the status LED DB-9 cables and insert a new set of 10 AA size batteries in the battery holder and secure the battery connector. The red LED should blink in about 6 to 7 min. The data is now ready to be transferred to the USB drive.	
6. After the RED led on the PACKMAN-B side has started to blink, press and hold the yellow button first and simultaneously press the green button. The red LED on the PCB should begin to glow. If the red LED on the PCB blinks three times, then there is an error with the USB drive. Ensure the USB drive is adequately fixed or replace the USB drive with another drive with FAT32 partition.	
7. If the red LED on the PCB lights continuously, then the data transfer process has started. Wait till the red LED on the PCB stops glowing. The data generated is high, especially after 8-hour flights, and the process will take a lot of time depending on the data frequency rate set for the sensors. The figure below shows the file transfer in progress with the red LED glowing.	
8. Once the red LED has stopped glowing, it is safe to disconnect the battery terminal and remove the USB drive from PACKMAN-B. The data then can be viewed on a PC. Disconnect the DB-9 LED pin. Connect the USB cable back to the Raspberry Pi. Put back the PCB with buttons in the Styrofoam slot and put back the insulation material on top of it and close the PACKMAN_LID. Now connect the DB-9 LED cable and close the Styrofoam box. The data in the PACKMAN-B is automatically erased after the contents are copied to the USB drive.	

## 8 Validation and Characterization

**Fig. 10:**
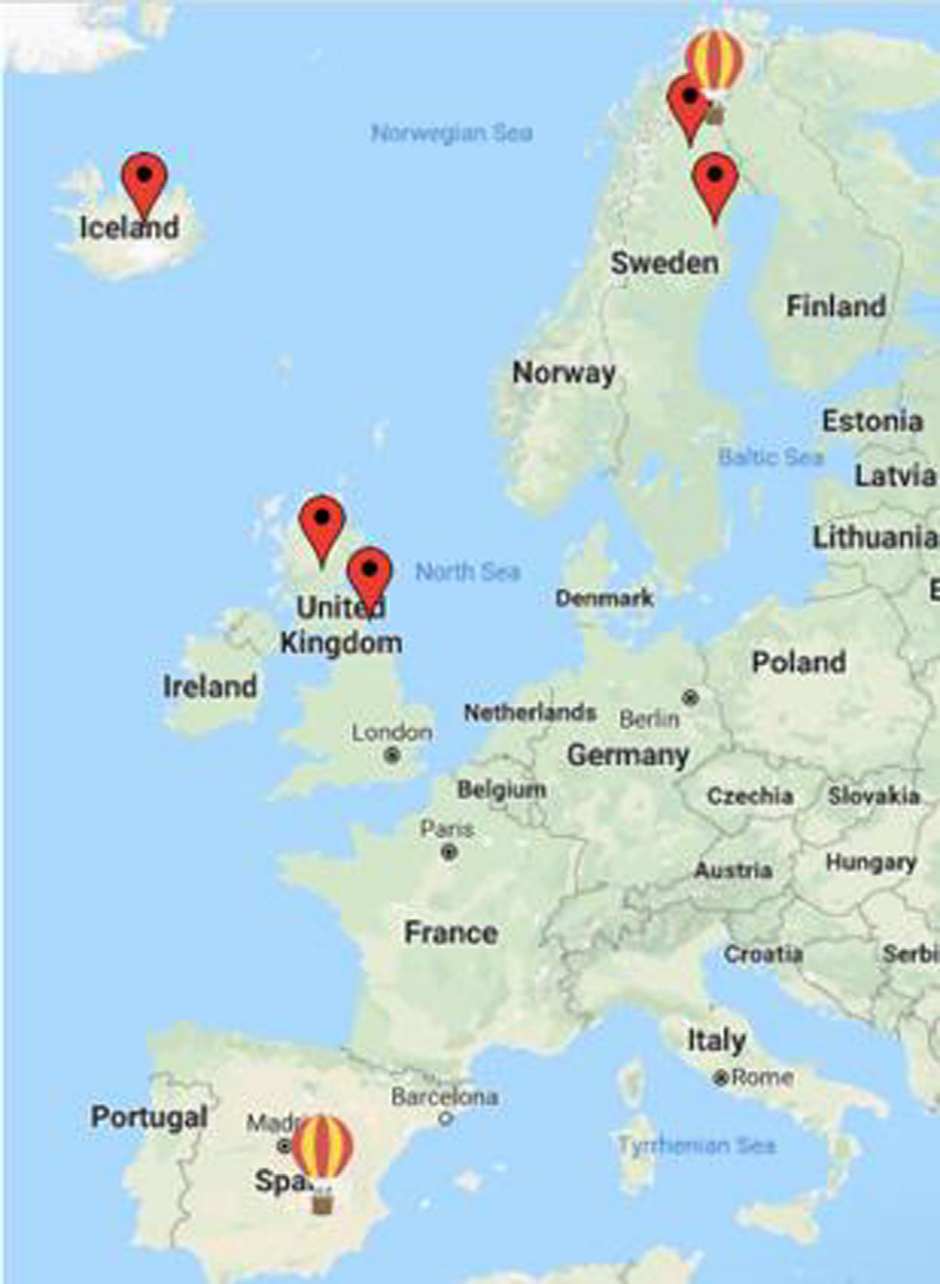
Map of PACKMAN installation sites and stratospheric balloon field campaigns.

PACKMAN was developed and tested in three campaigns, one within the Dark Matter Research facility, Boulby Mine, in cooperation with the UK Center for Astrobiology, and two balloon campaigns with Zero2Infinity (Córdoba, Spain) and, Esrange (Kiruna, Sweden) with the support of the Swedish Space Corporation (SSC). Some of the results that demonstrate TRL8 for surface and balloon operation have been presented before [18], [21]. The data distribution concept has been presented in [22] and in the concept for the ESA/European balloon community [23]. Fig. 10 shows the map of PACKMAN units operating sites.

The operating sites of PACKMAN are listed below in Table 6.

**Table 6:** PACKMAN deployment locations.

Location	Geographic Coordinates	Altitude
Space campus LTU, Kiruna, Sweden	67.84 °N, 20.41 °E	390 m
LTU Main campus, Luleå, Sweden	65.62 °N, 22.14 °E	15 m
Boulby Mine, Cleaveland, United Kingdom	54.56 °N, 0.82 °W	93 m
The University of Edinburgh, United Kingdom	55.94 °N, 3.19 °W	98 m
The University of Akureyri, Iceland	65.68 °N,18.12 °W	23 m
Cordoba airport, Cordoba, Spain	37.84 °N, 4.84 °W	90 m–27 km
Esrange Space Center, Kiruna, Sweden	67.88 °N, 21.12 °E	328 m–27 km

Some of the scientific observations made from PACKMAN are presented below, where the data was disseminated by PACKMAN’s during its operation at stratospheric altitude and subsurface levels of 1.1 km below the surface of the Earth.

### 8.1 PACKMAN-B

**Fig. 11:**
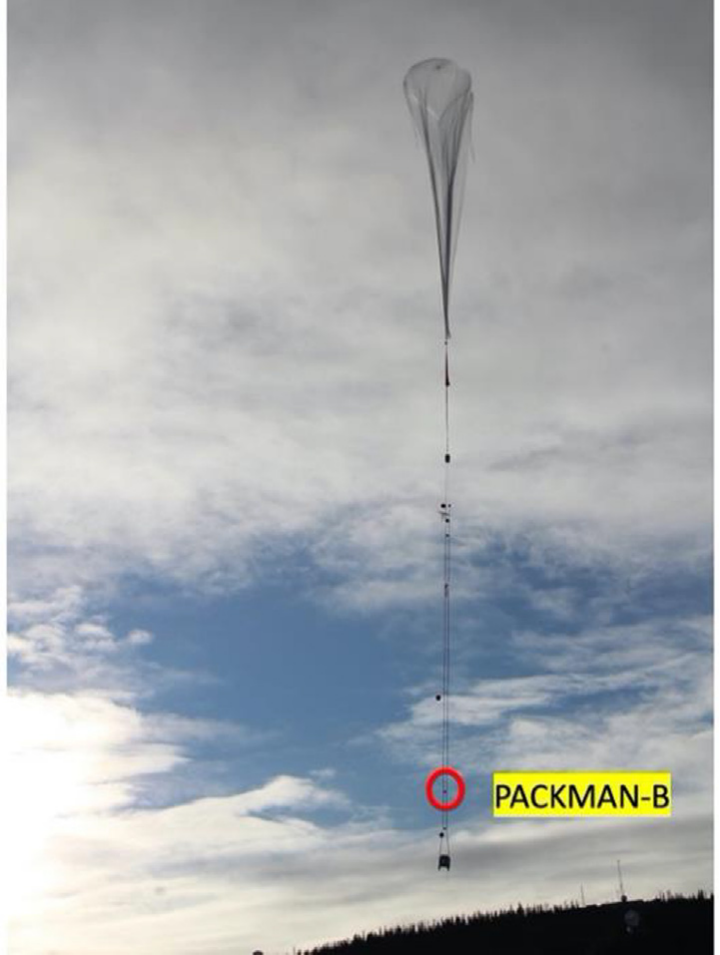
PACKMAN-B payload attached to the tether of the stratospheric balloon during its launch from Esrange Space Centre, Kiruna.

PACKMAN-B had been flown twice on stratospheric balloon campaigns as a piggyback payload with an approximate flight time of around 8 h. The maximum altitude reached during both the launches was approximately 27 km with temperatures reaching −47 °C. The maximum altitude reached during both the launches was approximately 27 km. The first flight took place at Cordoba Airport, Spain in collaboration with Zero2Infinity company and through the way up the Geiger tubes recorded a steady increase in radiation levels measured in particle Counts Per Minute (CPM). The rise was also observed during the second flight, which was from Esrange Space Centre in Kiruna 67.88 °N, 21.12 °E launched by Swedish Space Corporation (SSC). The Fig. 11 shows the PACKMAN-B payload attached to the tether of the stratospheric balloon as a standalone payload during the launch from Esrange Space Centre, Kiruna.

**Fig. 12:**
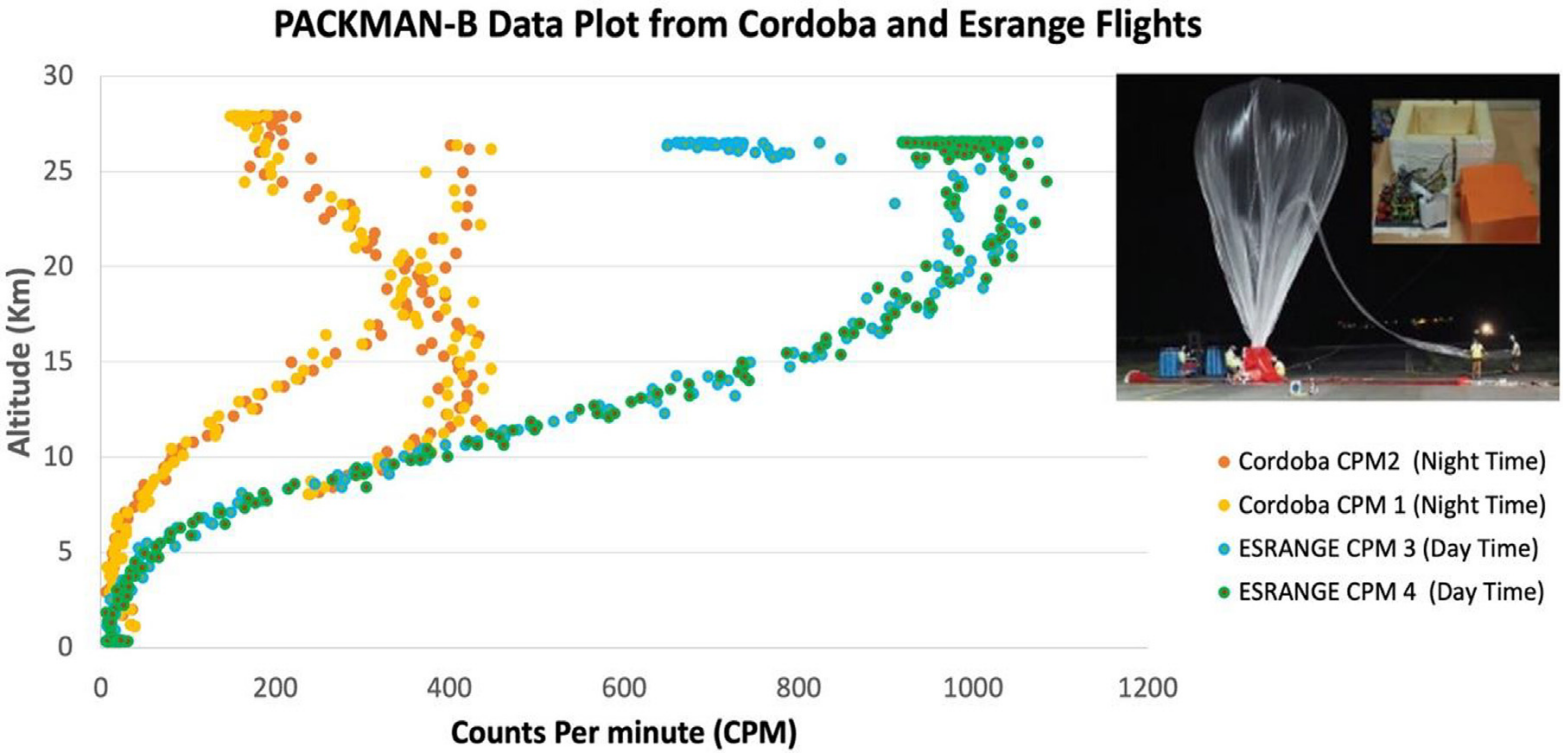
PACKMAN-B Geiger particle counts (CPM) against altitude. The yellow and orange dots indicate the CPM measurements during the flight from Cordoba, and the green and blue dots indicate the CPM measurements during the flight from the Esrange space centre. The inset image shows the PACKMAN-B payload during the Cordoba Flight campaign.

The rise in particle counts observed is due to the Pfotzer Maximum phenomenon, where there is an increase in the incoming secondary radiation levels reaching the lower layers of the atmosphere. The secondary radiation is produced when the primary cosmic rays reaching Earth, interact with the upper atmosphere and this secondary radiation intensify at lower heights around 15–20 km depending on latitude [24], where the loss effects balance the secondary radiation. This effect was observed by direct measurement of ionization at various altitudes in the atmosphere by Erich Regener and George Pfotzer [25], [26], [27]. The point at which the maximum radiation is measured is called the Regener-Pfotzer maximum (RP-max). Below this point, the secondary radiation intensity is gradually reduced due to absorption and decay processes [28], [29]. The Fig. 12 below, shows the plot of the radiation levels measured in CPM against altitude with the Pfotzer Maximum observed during both the flights. Similar studies of Pfotzer Maximum have been described in [30]. In PACKMAN, we are more interested in studying the collective particle dose rates and not in identifying the type of particles and their constituent energies. The instrument is meant to be light and autonomous, so that it can be easily mounted on balloons and give information about the vertical profile, its diurnal and seasonal variability. Future studies may use more specific high-cost instrumentation, in dedicated campaigns, as designed by geospace and ionospheric researchers. But this kind of instrumentation may be useful to provide a first assessment.

### 8.2 PACKMAN-S

**Fig. 13:**
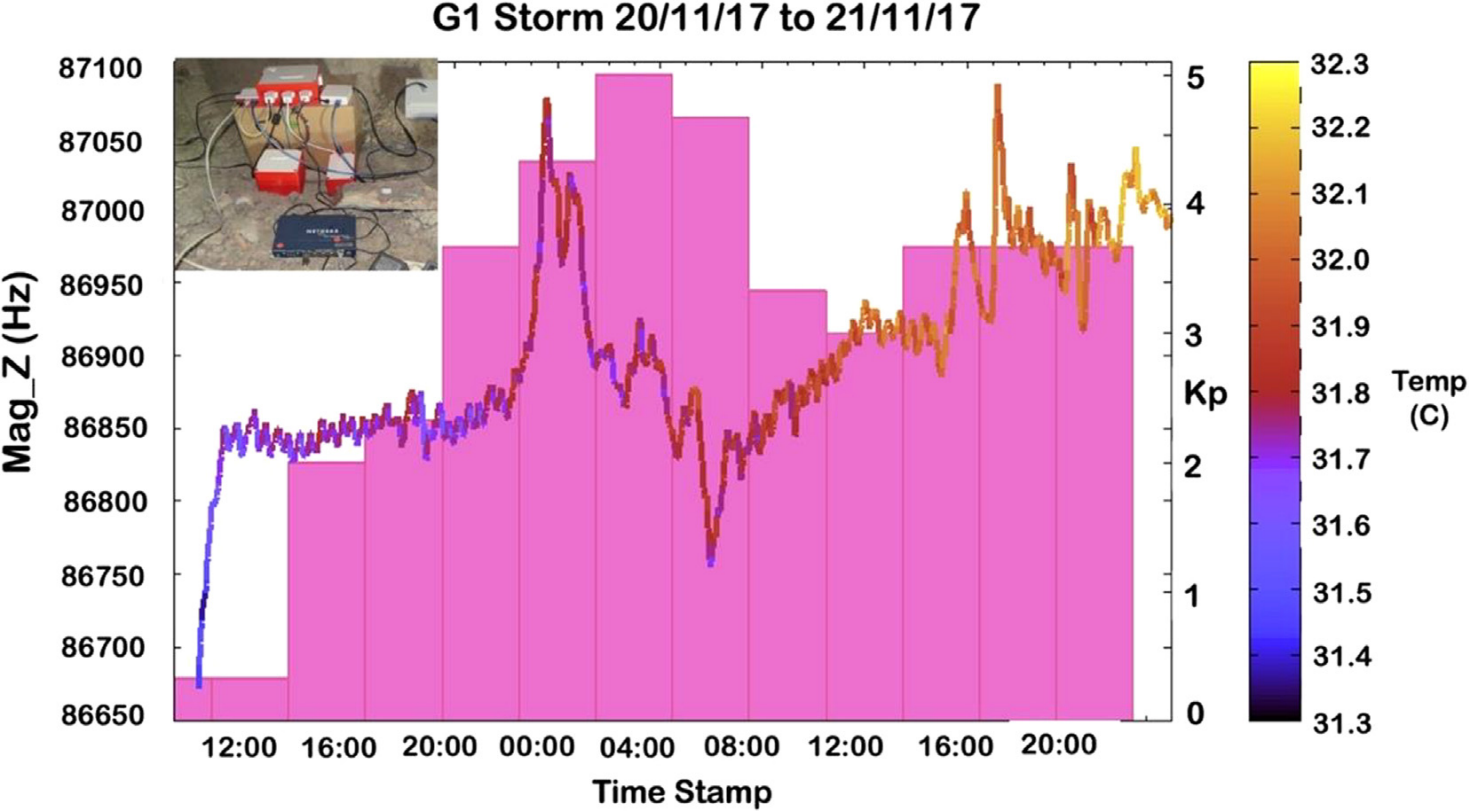
PACKMAN Magnetometer-Z response to G1 geomagnetic storm with temperature in the colour map and a bar graph showing the Kp indices. The inset image shows the PACKMAN-S operating in the Boulby mine, 1.1 km below the surface of the Earth.

The fluxgate magnetometers used in PACKMAN provide a frequency output in the range of 120 KHz to 50 kHz depending upon the magnetic field changes. The magnetometers are not calibrated to absolute magnetic field levels. The fluxgate magnetometers provide an almost linear response with change in magnetic field with a non-linearity of about 5.5%. During operation on a geomagnetically quiet day, an uncertainty in measurement of up to ± 15 nT could be observed in the readings [18]. The fluxgate magnetometers are very sensitive to temperature fluctuations and stray magnetic fields and would need a very stable environment to calibrate the frequency output to absolute magnetic field. The limitation in such facility required deferred from calibrating the fluxgate magnetometer and is not in the scope of this article. Future studies will be devoted to calibrating uncertainties, but the purpose of this article is to describe the fabrication of a low-cost instrument with COTS components. The manuscript provides details about the construction and operation of PACKMAN for the readers of this journal. The magnetometers are sensitive, translating a 1 nT magnetic field change to 1 Hz output, such that we could observe the changes in the frequency output during different conditions of geomagnetic activity. The geomantic activity is measured using a Kp index. In the plot shown in Fig. 13, the continuous fluxgate magnetometer response to G1 geomagnetic storm (Kp ∼ 5) that occurred between 20th November 2017 and 21st November 2017 is shown. The colour heat map, overlayed on the magnetometer measurement, indicates the temperature at the time of measurement. A stable temperature is one of the essential criteria for shallow magnetic field sensing as fluxgate magnetometers are very sensitive to the temperature fluctuation. Also, the magnetometers have an uncertainty in measurements up to ± 15 nT. The Styrofoam box with the thermal isolation padding provides a stable temperature environment for the fluxgate magnetometers of PACKMAN. The Kp values indicated have been taken from the GFZ Postdam Data Archive from Helmholtz Centre, Postdam.

**Fig. 14:**
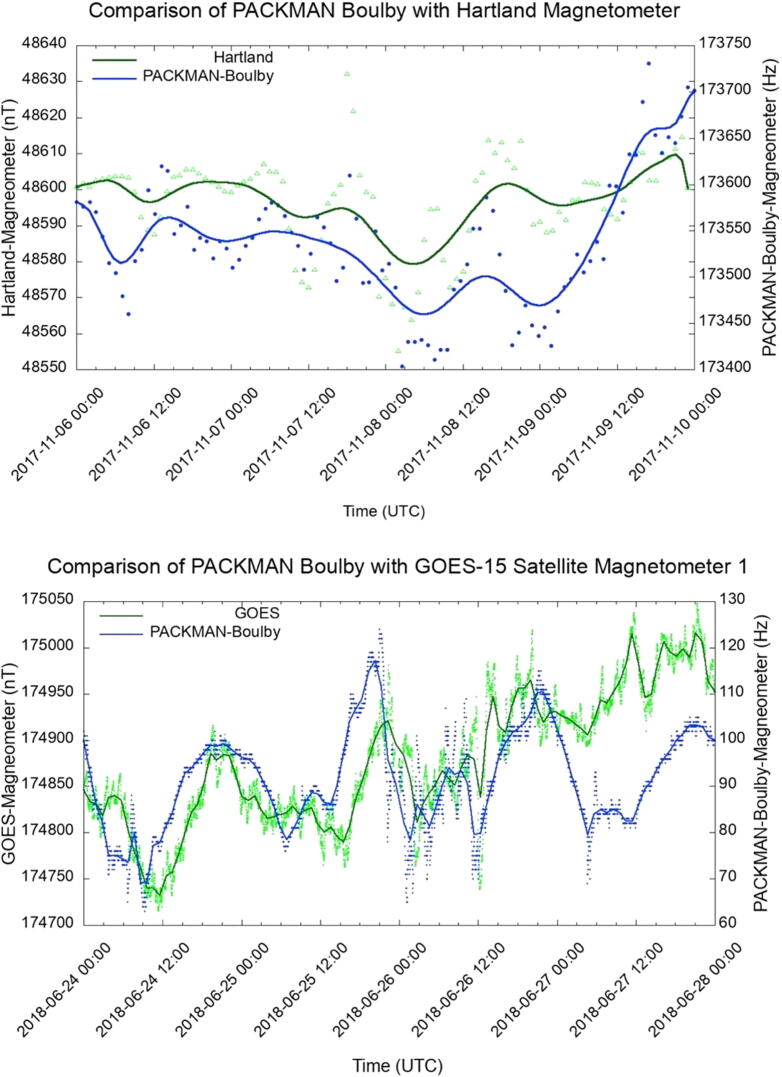
Comparison of the magnetic field measurements from the PACKMAN-S Boulby against the Hartland magnetic field observatory (Top) and GOES satellite data (Bottom).

The performance of the magnetometers in PACKMAN have been compared with the state-of-the-art magnetic observatories and satellite data. The magnetic field responses of PACKMAN-S operating at the Boulby mine, 1.1 km below the surface of the Earth has been compared with the magnetic field observations from Hartland station magnetometers which are situated at 50.995 °N, 355.516 °E, 95 m above sea level and is operated by the British Geological Survey. The plot in Fig. 14 (top) shows the correlation between the magnetic field perturbations recorded by the magnetometers in PACKMAN and the Hartland Magnetic observatory magnetometers during an increased solar activity with a Kp ranging between 4 and 6 (G2 storm) during the measurement period from 6th to the 9th of November 2017. The plot Fig. 14 (bottom) shows the comparison of the readings obtained from the PACKMAN-S operating in the Boulby mine with the GOES-15 Magnetometer 1. The readings (fitted with Bezier curves) are taken during the G1 storm that occurred between 24th and 27th of June 2018. Although GOES-15 is operating far from the Earth, and its measurements are dominated by magnetospheric processes, we include here some comparison to illustrate the different timescales of the variability of both magnetic measurements. Also, this is interesting to illustrate that portable instruments such as PACKMAN may be updated in the future to operate on CubeSats or on landed platforms on the Moon.

**Fig. 15:**
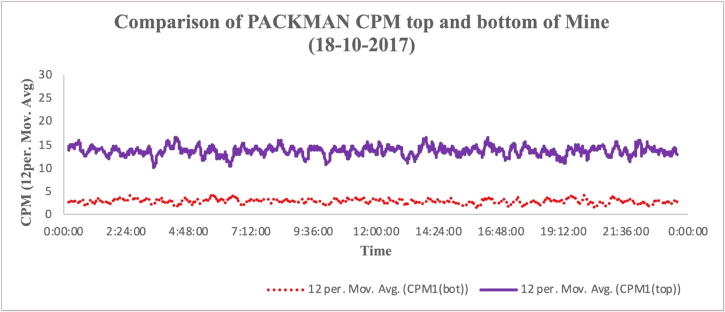
Figure showing the lower background radiation experienced in Boulby Mine (dots) compared with surface particle flux (solid line), validating the instrument in a ‘quiet’ radiation environment.

The particle response in subsurface environments was analysed with the PACKMAN-S operating down in the Boulby mine, 1.1 km below the surface of the Earth. The experiment was performed during the Mine Analog Research (MINAR) program at the Boulby Mine, UK [21]. The readings from the Geiger counters of PACKMAN-S unit operating on the surface of the mine was compared with the reading from the Geiger counters of PACKMAN-S unit operating below in the mine. The Fig. 15 shows the difference in particle counts observed. A 12-minute moving average has been taken to smoothen the plot. The radiation ‘quietness’ of the mine owing to the kilometre of crust that shields the mine tunnels from the background radiation can be observed.

A more detailed scientific analysis of the observations made by PACKMAN is discussed in [18], [31] where the data disseminated is compared to geomagnetic events of varied magnitudes.

## 9 Conclusion

The PACKMAN instrument is designed to be a low-cost, robust instrument to investigate space weather phenomena at varying latitudes and altitudes. The robustness of the instrument along with the TRL8 maturity, has been validated through stratospheric balloon launches in campaigns with Zero2Infinity (Córdoba, Spain) and, Esrange (Kiruna, Sweden) with the support of the Swedish Space Corporation (SSC) and deployment in the Boulby Mine, in cooperation with the UK Center for Astrobiology, operating at 1.1 km below the surface of the Earth. Also, the data from PACKMAN has been compared with the cutting edge sophisticated magnetic observatories and satellites which justifies the scientific maturity of the instrument. The use of COTS components ensures easy availability and parts can be replaced with suitable alternatives, should there be a need to upgrade with wear and use. Replacement of the components with appropriate options ensures that the data is repeatable and comparable with the other PACKMAN units. The open-access data approach from PACKMAN space weather observations can be useful for a wide range of fields apart from research, including aviation, infrastructure and telecommunication, as well as for education and outreach. A network of such open-source PACKMAN units operating around the world would help to fill the missing gap of information regarding the amount, energy, time variability, and type of space radiation that reaches the lower layers of the atmosphere, as well as on its geographic and altitude distribution and the implications on infrastructures and climate. Through this paper, the public can build PACKMAN units from scratch with minimal resources and contribute to the understanding of global space weather phenomena.

## Declaration of Competing Interest

The authors declare that they have no known competing financial interests or personal relationships that could have appeared to influence the work reported in this paper.
